# The Pitaya Flower Tissue’s Gene Differential Expression Analysis between Self-Incompatible and Self-Compatible Varieties for the Identification of Genes Involved in Self-Incompatibility Regulation

**DOI:** 10.3390/ijms241411406

**Published:** 2023-07-13

**Authors:** Zhouwen Wang, Meng Wang, Yi Ding, Tao Li, Senrong Jiang, Shaoling Kang, Shuangshuang Wei, Jun Xie, Jiaquan Huang, Wenbin Hu, Hongli Li, Hua Tang

**Affiliations:** 1 Sanya Nanfan Research Institute of Hainan University, Sanya 572025, China; 2 Hainan Key Laboratory for Sustainable Utilization of Tropical Bioresources, College of Tropical Crops, Hainan University, Haikou 570228, China; 3 Tropical Crops Genetic Resources Institute, Chinese Academy of Tropical Agricultural Sciences, Haikou 571700, China

**Keywords:** pitaya, self-incompatibility, transcriptome analysis, differential gene expression, *S-RNase*

## Abstract

Self-incompatible pitaya varieties have low fruit-setting rates under natural conditions, leading to higher production costs and hindering industrial prosperity. Through transcriptome sequencing, we obtained the 36,900 longest transcripts (including 9167 new transcripts) from 60 samples of flowers. Samples were collected pre- and post-pollination (at 0 h, 0.5 h, 2 h, 4 h, and 12 h) from two varieties of pitaya (self-compatible Jindu No. 1 and self-incompatible Cu Sha). Using the RNA-Seq data and comparison of reference genomes, we annotated 28,817 genes in various databases, and 1740 genes were optimized in their structure for annotation. There were significant differences in the expression of differentially expressed genes (DEGs) in the pitaya stigmas under different pollination types, especially at the late post-pollination stage, where the expression of protease genes increasedal significantly under cross-pollination. We identified DEGs involved in the ribosomal, ubiquitination-mediated, and phyto-signaling pathways that may be involved in pitaya SI regulation. Based on the available transcriptome data and bioinformatics analysis, we tentatively identified *HuS-RNase2* as a candidate gynogenetic S gene in the pitaya GSI system.

## 1. Introduction

In nature, flowering plants have developed a reproductive isolation mechanism, self-incompatibility (SI), to prevent inbreeding and species degradation, in order to maintain the genetic diversity of their species. Specifically, this means a (type of) SI that has complete flowers and can form normal female and male gametes, but which lacks the ability to set fruit after self-pollination [1,2]. Plants have evolved two different SI systems: sporophyte SI (SSI) and gametophyte SI (GSI) [3]. In SSI systems, pollen grain non-affinity is determined by the diploid genome of the parent. The S-locus receptor kinase (SRK) is located on the plasma membrane of the pistil stigma, and once pollinated, it is mutually recognized with S-locus cysteine-rich protein (SCR), which is deposited by the anther tapetum layer onto the pollen. When SCR and SRK belong to the same S genotype, specific interactions cause SRK to undergo autophosphorylation and activate downstream signaling, resulting in SI [4]. In GSI systems, the gametophyte S genotype determines the SI response, where the growth inhibition of pollen with S genotypes that are identical to the pistil mainly occurs in the style, which is based on the S-RNase and Ca^2+^ SI systems [5,6]. Of these two systems, the S-RNase-based GSI system can be traced back 90 million years [7,8]. It was first found in the flowering tobacco of Solanaceae [7,8], and then in Rosaceae [9], Plantainaceae [10], and Rutaceae [1,11,12]. This system is controlled by two genes closely linked at the S locus: the pistil S gene and the pollen S gene. Each family uses members of the T2/S-type ribonuclease (T2/S-RNase) gene family (*S-RNase*) as pistillate components [4,13,14], as well as several S-linked F-box-containing proteins as pollen components of self-pollen recognition and rejection responses (named S-locus *F-box* (*SLF*) or S-locus *F-box* brother (*SFB*)) [15,16]). Interaction of pistil S-RNase and pollen tube SLF (cytoplasm) can only happen after all pistil S-RNases enter (all) the growing pollen tubes, however, the SLF is unable to recognize its own S-RNase. Therefore, SI occurs. In the GSI system, S-RNase acts as a cytotoxin, degraded pollen tube RNA, while the SLF in the pollen of other flowers acts as a “detoxifier”. As such, the S-RNase will not degrade, thus exerting its cytotoxicity and leading to pollen RNA degradation, eventually resulting in the inhibition of pollen protein synthesis [7,8,9,10,11]. Typically, these recognition and rejection responses are manifested such that the pollen landing on the stigma can germinate normally; however, in the style, self-pollen is rejected, and the pollen tube cannot grow normally, resulting in the inability to fertilize [17].

Pitaya, also known as dragon fruit, belongs to the family of Cactaceae and includes two genera (one is *Hylocereus*, the other is *Selenicereus*). The main cultivated types are *H. polyrhizus, H. undatus,* and *S. megalanthus*—which is a monoecious plant belonging to the cactus family [18,19]. Self-incompatible varieties of pitaya show typical GSI. By observing the mating systems of the genera *Hylocereus* and *Selenicereus*, self-pollinations in *H. polyrhizus* resulted in inhibition of pollen tube growth at the ovary, in which SI S-RNase could prevent the growth of pollen tubes resulting in fertilization failure [20]. The discovery of the pistil S gene in the Rosaceae family of lentils and plums suggests that SLF is involved in ubiquitination-mediated protein degradation by acting as a substrate recognition subunit in the ubiquitin ligase E3, which mediates the ubiquitination of S-RNase through the 26S ubiquitin lysosome, which leads to the recognition reaction [21]. The mutual recognition of S-RNase and SLF may be well explained by the “synergistic non-self-recognition model”. Each S haplotype encodes an SLF that recognizes the allozygous S-RNases but is unable to degrade the ego-type S-RNases, whereas a single SLF recognizes only one type of S-RNase; thus, multiple *F-box* genes at the S locus work together to recognize all ego-type S-RNases, as well as to ubiquitinate and degrade them, thus allowing normal pollen tube growth. Conversely, if multiple *SLF*s encoding pollen tubes have the same S genes as *S-RNase*, then the pollen tube does not grow normally and shows SI when these S-RNases behave as toxic proteins. In addition, the polymorphism of multiple *SLF*/*SFB* genes is significantly higher than that of *S-RNase* genes, which fully ensures that all types of heterozygous S-RNases can be recognized and degraded [22].

During the agricultural cultivation of pitaya, the pollination and fruiting of most self-incompatible varieties are limited by short flowing time and high manpower cost due to its following characteristics. (1) Pitaya flowers bloom greatly and simultaneously for one night, with each flower lasting only one night before wilting. Moreover, there are few insects around at night (for example, bees), resulting in a low seed-setting rate of self-incompatible varieties under natural conditions; (2) Artificial auxiliary pollination is necessary [23], and it not only requires pollination technology of workers, but also is affected by climate, environment, and time [24], which greatly increase the production cost for growers [25].

In this study, we selected two pitaya varieties ‘Jindu No.1’ (self-compatible) and ‘Cu Sha’ (self-incompatible) as the materials for self-pollination and cross-pollination, respectively. Mature pollen and non-pollinated stigmas (0 h) and samples of pollinated stigmas from both varieties at 0.5 h, 2 h, 4 h, and 12 h were sampled and used for RNA-seq experiments to compare gene expression between SC and SI pollinations. We compared, via RNA sequencing, the gene expression patterns during self-pollination and cross-pollination in these mature pollen samples and pollinated pistils of both cultivars. We identified novel pitaya transcripts from one candidate *S-RNase* gene and several transcripts derived from different candidate *SLF*/*SLB* genes. These findings are important contributions to the study of the molecular mechanism of SI responses in pitaya. This work will help to identify and select self-compatible varieties of pitaya and promote the prosperity of its industry.

## 2. Results

### 2.1. Overview of Transcriptome Sequence Analysis and Alternative Splicing (AS) Events

We sequenced the cDNA libraries of stigmas and pollens from two pitaya varieties with Illumina high-throughput sequencing technology, among which, the stigmas that were included were unpollinated, self-pollinated, and cross-pollinated stages. Analysis of all transcriptome datasets revealed contamination with symbiotic viral RNA sequences, mainly *Schlumbergera* virus and *Cactus* virus transcripts (Supplementary Materials, Tables S1 and S2). We filtered our datasets to remove all viral sequences. The percentage of Q30 bases was not less than 91.79%, and the sequence comparison efficiency was 62.72–97.27% when compared with the specified reference genome (http://www.pitayagenomic.com/download.php (accessed on 5 June 2022) (Supplementary Materials: Table S3). After examination, the reads were homogeneously distributed over all the expressed genes. The number of double-ended reads at different distances between the start and end points of the reference genome ranged from 0–200,000, the length of the inserted fragment ranged from 0–800 bp, and they had an overall normal distribution. In this study, the number of DEGs obtained by sequencing tends to be saturated; thus, the mapped data are sufficient for subsequent analysis.

**Figure 1:**
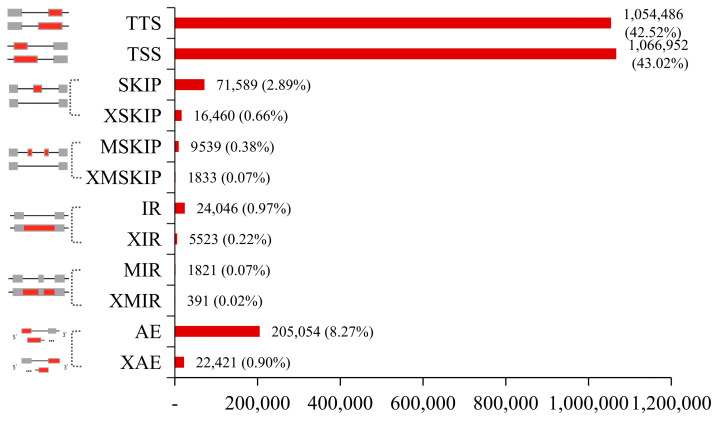
The AS events of all transcripts. The AS events in the 60 samples, showing the number and percentage of each type. TSS: Alternative 5′ first exon (transcription start site), i.e., the first exon splicing; TTS: alternative 3′ last exon (transcription terminal site), the last exon splicing; SKIP: skipped exon (SKIP_ON, SKIP_OFF pair), single exon skipping; XSKIP: approximate SKIP (XSKIP_ON, XSKIP_OFF pair), single exon skipping (fuzzy boundary); MSKIP: multi-exon SKIP (MSKIP_ON, MSKIP_OFF pair), multi-exon skipping; XMSKIP: approximate MSKIP (XMSKIP_ON, XMSKIP_OFF pair), multi-exon skipping (fuzzy boundary); IR: intron retention (IR_ON, IR_OFF pair), single intron retention; XIR: approximate IR (XIR_ON, XIR_OFF pair), single intron retention (fuzzy boundary); MIR: multi-IR (MIR_ON, MIR_OFF pair), multi-intron retention; XMIR: approximate MIR (XMIR_ON, XMIR_OFF pair), multi-intron retention (fuzzy boundary); AE: alternative exon ends (5′, 3′, or both); and XAE: approximate AE variable 5′ or 3′ end (fuzzy boundary).

StringTie was applied to assemble the mapped reads generated by Hisat2. AS profile was employed to predict AS events in each sample and sort them into 12 types (Figure 1), which mainly occurred at the 5′ and 3′ ends of the pre-mRNAs. The alternative 5′ first exon (transcription start site, TSS) was the largest (1,066,952 events, 43.02%). The alternative 3′ last exon (terminal transcription site, TTS) was second only to TSS (1,054,486 events, 42.52%), while the approximate multi-IR (XMIR_ON, XMIR_OFF pair) had the smallest percentage of all AS events (391 events, 0.02%) [26]. The variable exon phenomenon appeared more frequently in the samples at all periods of stigma than in the pollen samples (Supplementary Materials: Table S4), which showed that the stigma was in a dynamic developmental process after pollination and evidently produced more mature mRNAs and increased the functional diversity of proteins.

### 2.2. Identification of Novel Genes and Functional Annotation

To identify the new transcripts, StringTie was used to splice the mapped reads. In comparison with the original genome, some of the reads were compared to the intergenic region and were able to splice the complete open reading frame, thus identifying the original unannotated transcribed region and complementing and improving the original genome annotation information. By filtering out the sequences that were encoding peptide chains that were too short (i.e., less than 50 amino acid residues) or contained only a single exon, we uncovered 9167 new transcripts, and 2935 new genes were annotated, with the largest number of 2792 annotated in the non-redundant protein (nr) sequence database. Thus, based on the 11 chromosomes and 860 scaffolds in the reference pitaya genome, we finally found that the 36,900 longest transcripts and the 28,817 genes were functionally annotated in nr, Swiss-Prot protein (Swiss-Prot), Gene Ontology (GO), Pfam, Clusters of Orthologous Groups (COG), clusters of orthologous groups for eukaryotic complete genomes (KOG), and the Kyoto Encyclopedia of Genes and Genomes (KEGG) databases. Thus, the function of the genes was analyzed. In addition, due to software or data limitations regarding the genome itself, we found that 1740 of the original annotations were supported by contiguous mapped reads outside the gene boundaries, thus their untranslated regions (UTRs) were extended upstream and downstream to optimize their structure.

**Figure 2:**
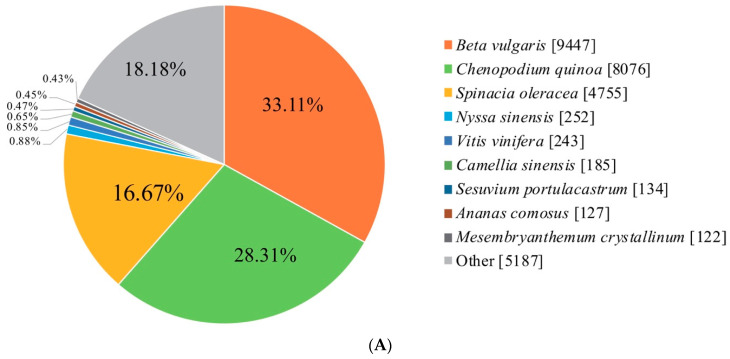

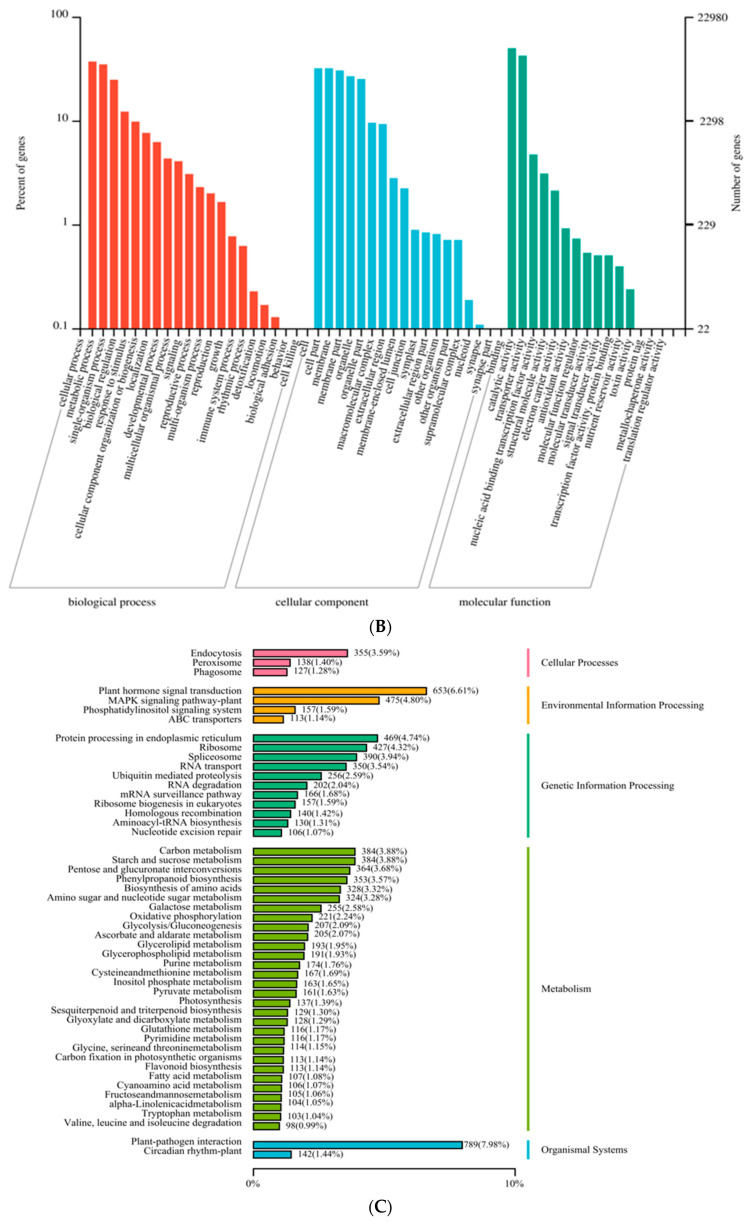
Functional annotation of all the transcriptome contigs. (**A**) Results and percentage of all genes annotated in the nr database. (**B**) Number and percentage of the transcripts in the GO classification. (**C**) Number and percentage of the transcripts in the KEGG classification.

In total, 28,529 transcripts (77.3%) were assigned to the nr database (Figure 2A), with *Beta vulgaris* (9, 447 genes; 33.11%), *Chenopodium quinoa* (8076 genes; 28.31%), and *Spinacia oleracea* (4755 genes; 16.67%) showing the largest number of hits. The GO annotation system revealed that 22,984 transcripts (62.3%) could be classified into three categories: “biological processes”, “cellular components”, and “molecular functions” (Figure 2B). The classification of cellular components of GO terms was mainly related to cells, membranes, and organelles, while the most abundant biological process GO terms were classified as “cellular processes”, “cellular components”, and “molecular functions”. The most abundant molecular function GO terms were classified as “binding” and “catalytic activity”. A total of 19,504 genes were annotated in the KEGG database (Figure 2C); specifically, in addition to the plant itself interacting with pathogens (ko04626, 789), the top four annotated KEGG terms were classified as “plant hormone signal transduction” (ko04075, 653), “MAPK signaling pathway–plant” (ko04016, 475), “protein processing in endoplasmic reticulum” (ko04141, 469), and “Ribosome” (ko03010, 427). Therefore, the genes annotated in the MAPK cascade signaling pathway are involved in regulating important plant growth and development processes.

### 2.3. Gene Expression Profile of Different Tissues

**Figure 3:**
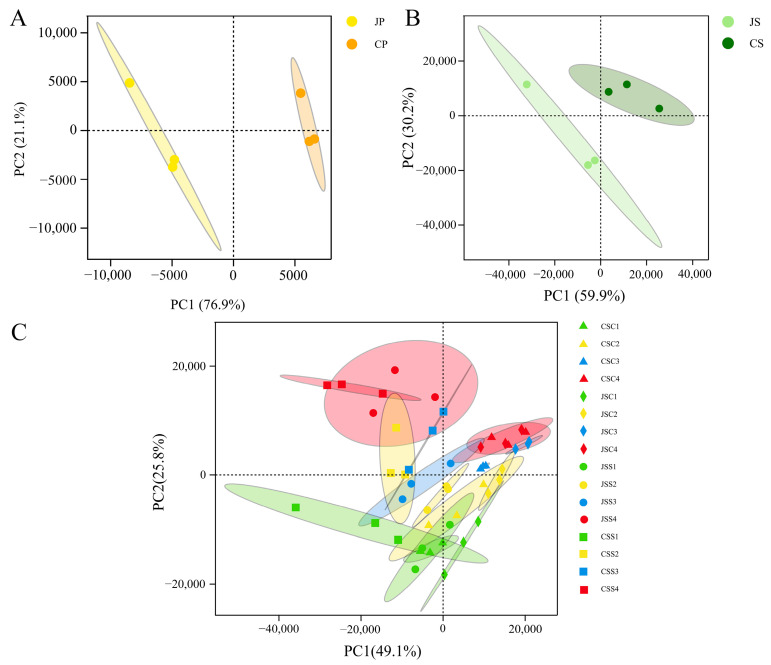
Expression analysis of all the transcripts. (**A**) Transcript expression levels for sixty samples, the y-axis indicates log_10_FPKM. (**B**,**C**) Principal component analysis of all the tissue samples from the FPKM data, colored according to different tissues and pollination types.

To understand the transcriptional dynamics of two types of pollen and two pollination types in pitaya, we measured the expression level of transcripts using the fragments per kilobase of transcript per million fragments mapped (FPKM) [27] method; then, we converted the FPKM values using log transformation (log_10_ (FPKM)). Principal component analysis (PCA) based on the expression of the different samples showed that all samples were within the 95% confidence interval, and there was a high similarity between the three biological replicate samples, indicating the high reliability of the transcriptional data (Figure 3B,C).

In the PCA analysis of both pollen samples (Figure 3A), principal component (PC) 1 (76.9%) was able to separate JP from CP. Similarly, in both unpollinated stigmas, PC 1 (59.9%) separated JS from CS (Figure 3B), showing significant differences between the two pollens and the stigmas themselves. In addition, each sample from different stigmas was clustered into 16 categories according to 2 pollination types and pollination times, where PC1 captured 49.1% of the variance of the data, indicating that genes in different groups differed significantly in transcript levels. During the 0.5–12 h post-pollination period, stigma samples from different treatments gradually diverged with time, and then—finally—the PC2 (25.8%) separated all samples at 0.5 h and 12 h post-pollination, indicating that the transcription products of the two pitaya genotypes changed significantly with increasing pollination time (Figure 3C). In this process, there was a clear separation between self-pollination (JSS, CSS) and cross-pollination (JSC, CSC), indicating that the transcripts were expressed in different patterns under different pollination types, especially in the late transcription period. PC1 completely separated the 12 h self-pollinated and hetero-pollinated stigma samples, indicating that after hetero-pollination, the stigma responds positively to different types of pollen and produces a large number of different transcription products.

### 2.4. Gene Co-Expression Analysis for Different Pollination Types

**Figure 4:**
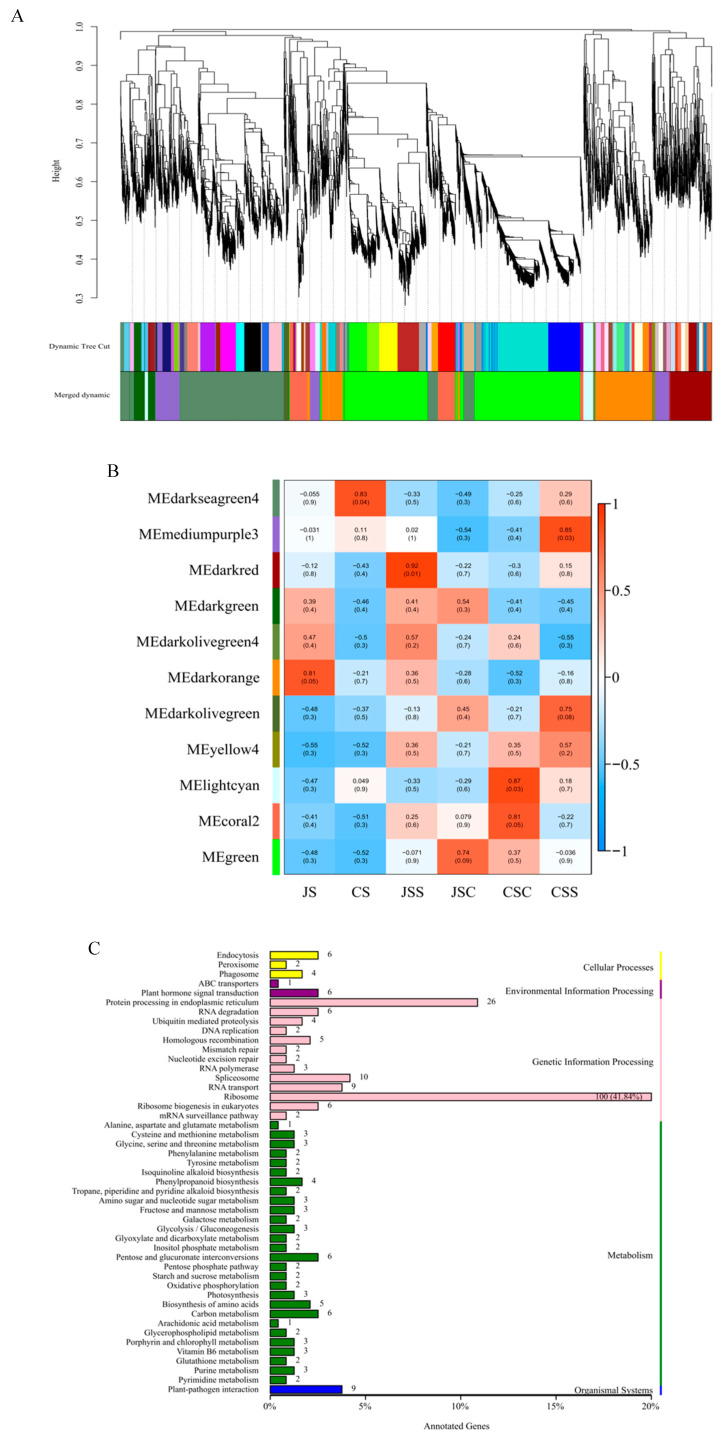
Transcript expression patterns of the six stigma samples. (**A**) Each functional module is represented by a different color, and the major branches after each clustering are represented by one color module. (**B**) Correlation heat map of the 11 modules and 6 classes of stigma. The values in the boxes indicate Pearson correlation coefficients and their *p* values, and ME refers to module eigengene. (**C**) The number and percentage of gene annotations to the KEGG pathway in mediumpurple3 modules.

To explore the changes in the transcription products of the two types of stigmas after self-pollination and cross-pollination, we clustered all stigma samples (JS, CS, JSS, JSC, CSC, and CSS) into six categories in order to find the genes expressing commonality that exist in different samples. For this, we constructed a weight-based gene co-expression network analysis (WGCNA) approach to explore the biological relationships and potential functions of stigmas under different pollination types. In this study, a total of 7911 filtered genes (FPKM > 1) were used for the subsequent analysis. At completion, 11 modules were obtained in the 6 classes of the stigma samples (Figure 4A), with the number of genes within each module ranging from 33 (darkolivegreen4) to 2650 (green).

Based on the correlation criterion of r > 0.75, we evaluated the gene co-expression patterns of 11 modules and the 6 classes of samples (Figure 4B). The green module containing the highest number of genes (2650) was positively correlated with both stigmas (JSC, CSC) in the heterogeneous pollination mode, while in the light cyan module, it was highly correlated with CSC (*r* = 0.87, *p* = 0.03) but negatively correlated with JSC. Similarly, the darkseagreen4 module was positively correlated with self-incompatible stigmas (CS, CSS) under self-pollination and was also highly correlated with unpollinated self-incompatible stigmas (CS) (*r* = 0.83, *p* = 0.04), but it was negatively correlated with the remaining four samples. We also found that the clustering of genes under different modules may be related to gene function; for example, genes in the mediumpurple3 module (*r* = 0.85, *p* = 0.03) and genes in the dark red module (*r* = 0.92, *p* = 0.01) were highly correlated with two stigmas with different affinities (CSS, JSS) under the self-pollination method, respectively. In the mediumpurple3 module, we detected 239 differentially methylation region (DMR) genes annotated into 47 KEGG pathways (Figure 4C), of which 177 genes (74.06%) were enriched in the KEGG category for genetic information processing, with the ribosome pathway being enriched with the most genes (100, 41.84%). Meanwhile, only 43 (19.37%) of the 222 DMR genes detected in the dark red module were annotated with genetic information processing (Supplementary Materials: Table S5). Therefore, genes in the mediumpurple3 module may have more mRNAs decoded by the ribosome to produce proteins that are different from those in the dark red module and thus may be involved in the mechanisms associated with SI in response to pitaya.

### 2.5. Effect of Self-Pollination and Cross-Pollination on Stigma Gene Expression

**Figure 5:**
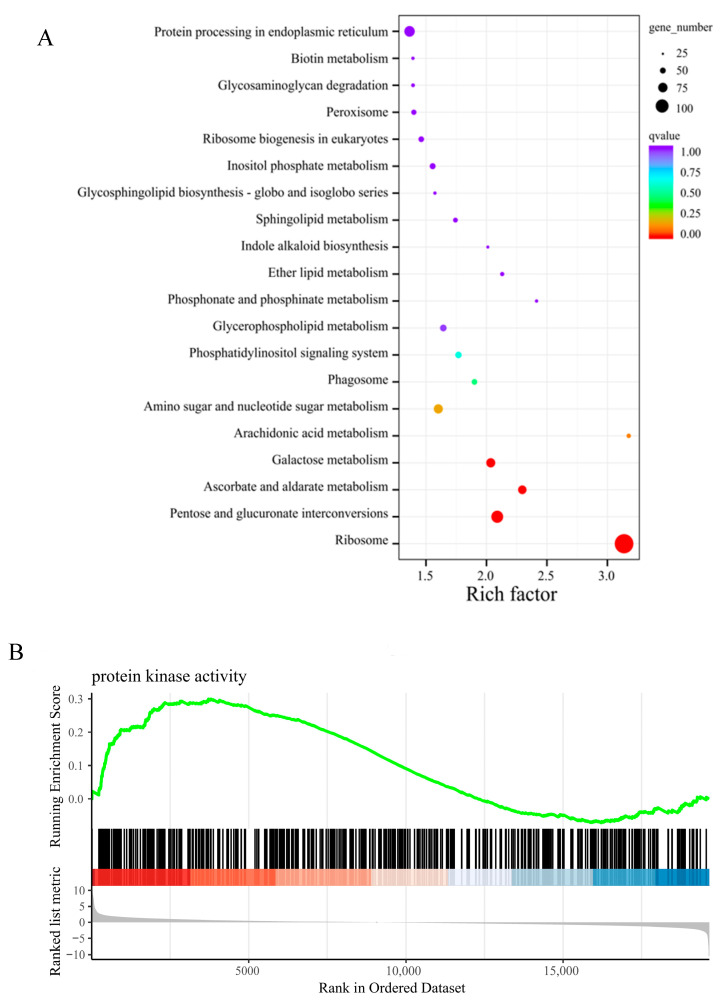

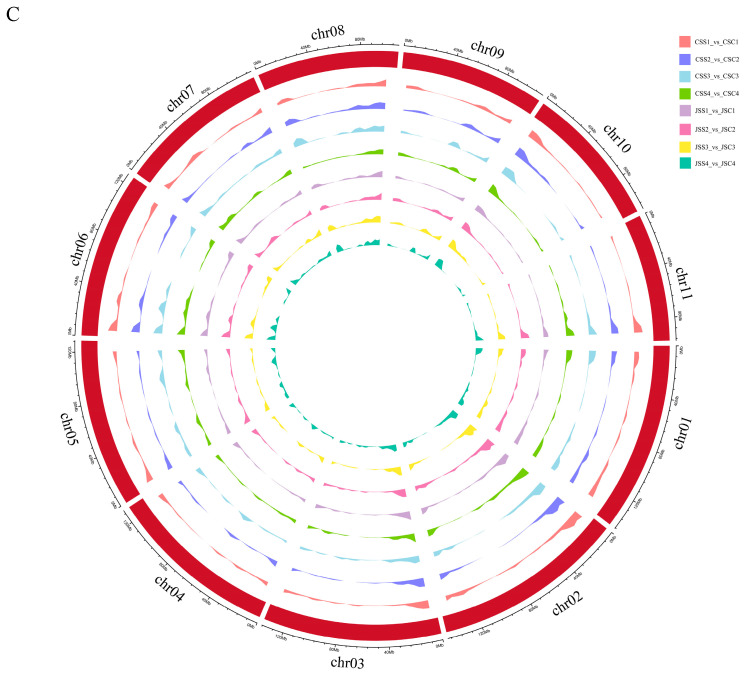
Expression pattern of the DEGs in the same type of stigma under two pollination types (CSS_vs_CSC and JSS_vs_JSC). (**A**) KEGG enrichment analysis of the DEGs. The circle represents the number of enrichment factors enriched in the KEGG database—the larger the circle, the more genes there are (the color represents the q value). (**B**) Gene enrichment analysis. The peaks in the upper dashboard plot appear at the front end of the sequenced gene set (ES values greater than 0) to indicate pathway upregulation and at the back end (ES values less than 0) to indicate pathway downregulation; the middle vertical line indicates the density of the gene arrangement, with red indicating that the gene is highly expressed in cross-pollination and blue indicating that the gene is highly expressed in self-pollination. (**C**) Differences in the CSC _vs_ CSS (outer four circles) and JSS_vs_JSC (inner four circles) methods 0.5–12 h after pollination localization of the exons in the genomic chromosomes. The area of the color indicates the abundance of the gene at that location.

The essence of the GSI system is that the RNase secreted by the mature pistil inhibits only those pollen tubes from plants with the same S-haplotype, preventing the elongation of the pollen tube. To investigate the changes in gene expression that changes in gene expression after self-pollination and cross-pollination on the self-compatible and self-incompatible stigma of pitaya, we analyzed the expression patterns of the 819 DEGs that are common to CSC_vs_CSS and JSS_vs_JSC. The KEGG enrichment analysis revealed the important roles of the differential genes in the ribosome, pentose, and glucuronate interconversions; the ascorbate and alternate metabolism; and the galactose metabolism pathways after cross-pollination (Figure 5A). The results of the constructed gene set enrichment analysis (GSEA) [28] showed that the GO terms of DEGs with two pollination types occurred mainly in molecular functions; in addition, the functional genes catalyzed more protease activity after heterogeneous pollination (Figure 5B).

In addition to the differential analysis at the gene level, the results of the differential analysis at the exon level [29] showed that most of the differential exons are distributed at the chromosomal ends of the genes, which is where the gene expression was active. However, the position and abundance of the differential exons change with pollination time. For example, in chr09, the outer four circles indicate the variation of the differential exons in the four stages of the Cu Sha samples under two pollination types. It was found that the position and abundance changed significantly at different times, which was different from the position of differential exons in the inner four circles of the Jindu No. 1 samples (Figure 5C).

### 2.6. Stigma Gene Expression Patterns at Different Time Points Post Pollination

**Figure 6:**
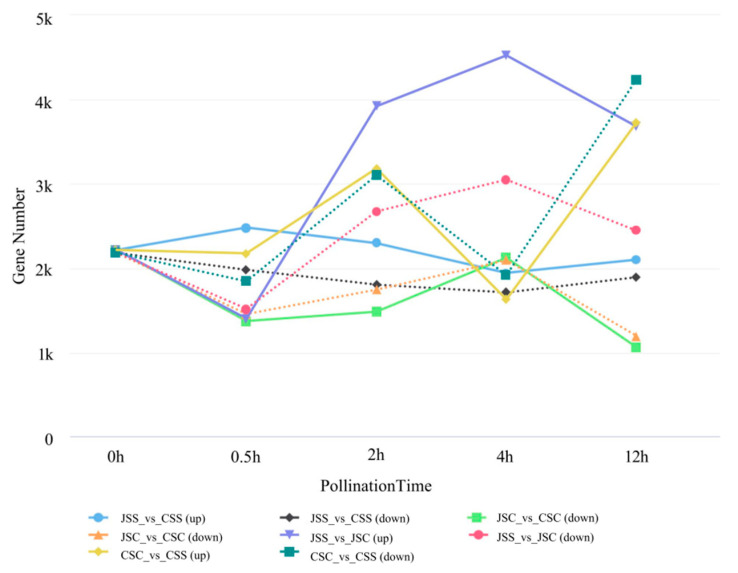
The change in the number of DEGs in all stigma samples, including unpollinated and 0.5 h, 2 h, 4 h, and 12 h after pollination.

DEGs after pollination were selected from the groups using thresholds of fold change ≥ 2 and FDR < 0.01 (Table 1, Figure 6). Regarding the stigma, there were significantly more DEGs in the self-pollinated than in the cross-pollinated groups after 0.5 h, 2 h, and 4 h of pollination, except for 4 h after pollination. In the stigma samples after self-pollination (JSS_vs_CSS), there were generally more upregulated DEGs than downregulated expressed genes in all periods, implying that more genes were expressed in self-incompatible stigmas than in self-compatible stigmas under self-pollination, while the opposite was also essentially true in the case of hetero-pollination (JSC_vs_CSC). To investigate the effect of the two pollination types on stigmas, we found that the number of DEGs gradually increased in both Jindu No. 1 (JSS_vs_JSC) and Cu Sha (CSC_vs_CSS) within 2 h after pollination. However, at 4 h after pollination, both showed completely opposite trends: the number of up- and downregulated DEGs in the pollinated stigmas of Cu Sha rapidly decreased and then rapidly increased at 12 h after pollination, which indicated that the two pollination types had a greater effect on the stigmas with different affinities at the later stages of transcription.

**Figure 7:**
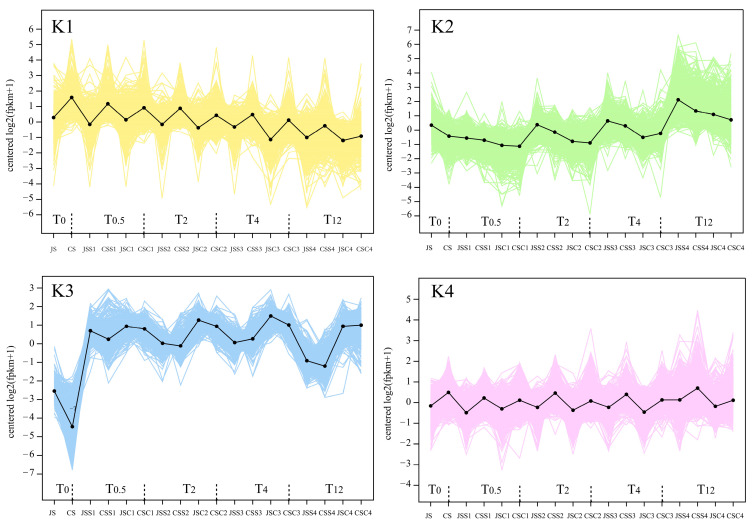

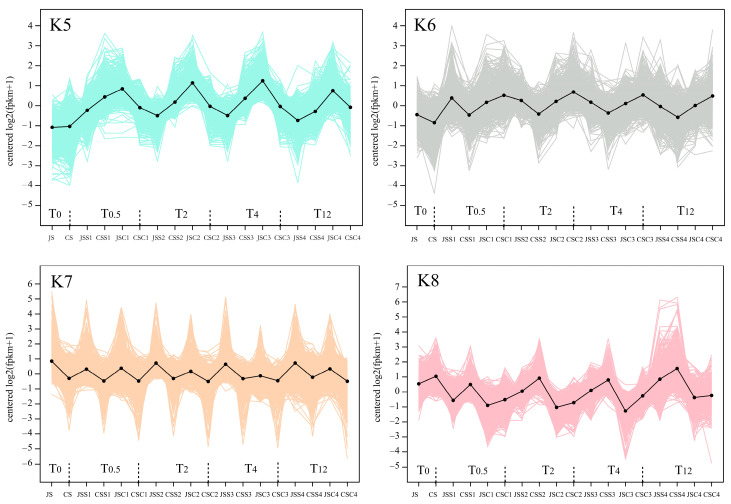
Analysis of the co-expression trends of DEGs in the stigmas of Jindu No. 1 and Cu Sha with the unpollinated, self-pollinated, and cross-pollinated stigmas at 0.5 h, 2 h, 4 h, and 12 h post-pollination. The *x*-axis indicates the different stigma samples, and the *y*-axis indicates the gene expression level, which is specifically expressed as log_2_(FPKM + 1). The DEGs pooled in each of the eight clusters were colored to form background lines, and the solid-colored lines indicate the overall trend.

To further investigate the development of stigmas at different time points after pollination in pitaya, our analysis of the co-expression trends of DEGs at each period showed that all DEGs were classified into eight categories (Figure 7). As pollination time increased, the expression of each transcript from cluster K1 (960) showed a slightly decreasing trend. Conversely, the expression of transcripts from cluster K2 (1198) gradually increased. In the K3 cluster (796), transcript expression showed a significant increase in each period after pollination, while transcript expression in the remaining clusters differed only in the different samples and was smoothly and differentially expressed in each period. The GO enrichment analysis of the differential transcripts from these eight clusters revealed that key differential transcripts were associated with a series of KEGG pathways, such as the ribosome, protein processing in the endoplasmic reticulum, and ubiquitin-mediated proteolysis. Notably, according to the production results, only the self-incompatible self-pollination (CSS) was eventually unfruitful, while the expression trends before and after transcription of each sample from cluster K8 (1558) showed that the expression in each period (CS–CSS4) was significantly higher in the case of self-pollination in self-incompatible varieties than in the other samples. In particular, a few genes showed a significant increase in expression at 12 h post-transcription, as seen by the background line. The genes clustered in the K8 cluster were not only associated with SI in pitaya but also with the final developmental phenotype of pitaya under each pollination method (Supplementary Materials: Figure S1).

### 2.7. Analysis of the DEGs Associated with the SI Response in Stigmas

**Figure 8:**
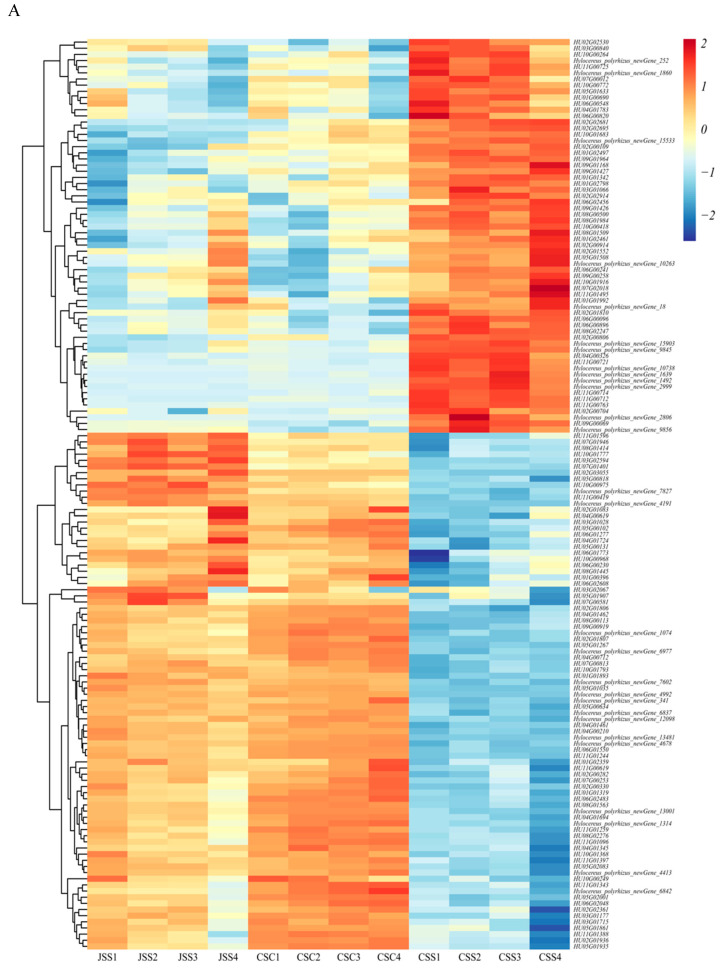

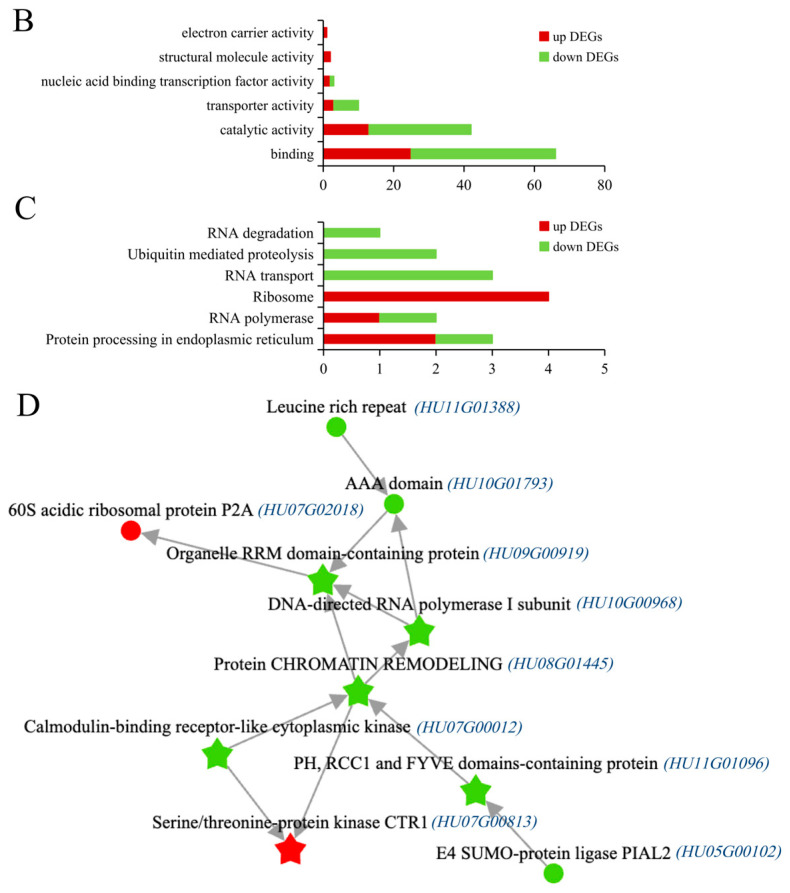
Expression patterns of the SI-related transcripts in the stigma. (**A**) Heat map analysis of the DEGs in the three types of stigma samples. (**B**) Comparison of the differences in the up- and downregulated genes enriched into GO primary taxonomic molecular functions. Red indicates upregulated DEGs and green indicates downregulated DEGs. (**C**) Comparison of the differences in the up-and downregulated genes enriched into the KEGG Genetic Information Processing pathway. Red indicates upregulated DEGs and green indicates downregulated DEGs. (**D**) Protein interactions of the key genes. In (**B**–**D**), red represents upregulated genes and green represents downregulated genes.

We screened 148 clearly annotated DEGs in self-pollinated (JSS_vs_CSS) and cross-pollinated (CSC_vs_CSS) stigmas. Their raw data were normalized for the mean and standard deviation (Z-core). The clustering heat map showed that 64 transcripts were upregulated, and 84 transcripts were downregulated in JSS_vs_CSS and CSC_vs_CSS (Figure 8A). Among the molecular functions classified at the GO level, we found 43 upregulated DEGs and 78 downregulated DEGs involved in binding (25, 41), catalytic activity (13, 29), transporter activity (3, 7), and nucleic acid binding transcription factor activity (2, 1) (Figure 8B). In addition, five DEGs were also annotated to protein processing in the endoplasmic reticulum (2, 1) and RNA polymerase (1, 1) in the KEGG pathway (Figure 8C). Notably, the upregulated genes *HU07G02018* and *HU09G01168* were enriched to the category of “structural molecular activity” in the GO database. In addition, they were annotated to ribosomes in the KEGG pathway as ribosomal 60S acidic ribosomal protein P2A and 60S ribosomal protein L6, respectively. The E4 SUMO-protein ligase PIAL2 (*HU05G00102*), pleckstrin homology (PH), regulator of chromatin condensation 1 (RCC1), and Fab-1, YGL023, Vps27, and EEA1 (FYVE)-domain-containing protein (*HU11G01096*), which is downregulated in SI, is a component of ubiquitin ligase E3, and is responsible for ubiquitin protein and substrate linkage (Figure 8C). The results of the selected 10 key proteins’ protein–protein interactions (PPIs) showed that calmodulin-binding receptor-like cytoplasmic kinase (*HU07G00012*), DNA-directed RNA polymerase I subunit (*HU10G00968*), and Organelle RRM domain-containing protein (*HU09G00919*)—which control plant growth, development, and protein synthesis—are key nodes, and the two proteins involved in ubiquitination-mediated interactions are also involved in sub-network interactions (Figure 8D). These DEGs provide candidates for subsequent studies.

### 2.8. Selection and Identification of the Pistil S-RNases Related to SI Traits

**Figure 9:**
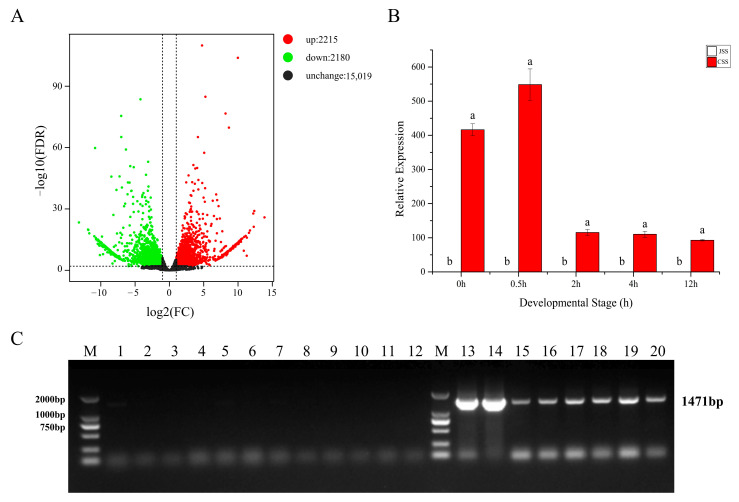
(**A**) Volcano plots of gene numbers in two types of unpollinated stigmas, with red dots representing DEGs with upregulated expression and green dots representing DEGs with downregulated expression. (**B**) Results of qRT-PCR of *HuS-RNase2* in two types of stigmas at 0.5 h, 2 h, 4 h, and 12 h after unpollinated, self-pollination. (**C**) The *HuS-RNase2* population validation in the genome of pitaya stems, M (marker): 2000 bp; 1–12: Jindu No. 1, Gui Honglong No. 1, Fu Guihong, Da Hong, Wuci Honglong, Shuang Se, Xiang Milong, Yan Wo, Mei Long, Di Long, Ruanzhi Dahong, Mi Xuanlong; M(marker): 2000 bp; 13–20: Cu Sha, Touxin No. 6, Bai Shuijing, Hong Shuijing, Aozhou Huanglong, Mi Bao, Wuci Huanglong, Hongpi Bairou. Different lowercase letters (a and b) indicate significant differences (*p* > 0.01).

To further screen for key genes responding to SI in pitaya, we identified the genes annotated with S-locus-associated ribonuclease (*S-RNase*) in the database. We retrieved two *S-RNase*s i.e., *HuS-RNase*1 (*HU11G00773*) and *HuS-RNase2* (*Hylocereus_polyrhizus_newGene_13356*), from the current transcriptome data. Using fold change ≥ 2 and FDR < 0.01 as the criteria, we mapped the significantly altered genes in the volcano map for JS and CS and screened 4395 DEGs (up: 2215, down: 2180) (Figure 9A). Interestingly, the differentially expressed gene set only had *HuS-RNase2* annotated, which is 1471 bp long and present in the two conserved regions of the RNase_T2 superfamily (cl00208) (Supplementary Materials: Figure S2), suggesting that this gene may be a key gene associated with SI in pitaya.

Moreover, we analyzed the expression patterns of the differential genes in two different types of stigmas at unpollinated and post-pollinated time points. The expression of *HuS-RNase2* was verified by qRT-PCR in the Jindu No. 1 (JSS) and Cu Sha (CSS) stigmas from different time points of self-pollination. The results showed that the gene was not expressed in all the Jindu No. 1 stigma samples, while it was expressed in all Cu Sha stigmas. Moreover, the expression of this gene was highest at 0.5 h after self-pollination in the Cu Sha stigmas, but this decreased with increasing pollination time (Figure 9B). We designed bidirectional primers based on the amino acid sequence of the *HuS-RNase2* transcript. Then, the gene was amplified using DNA from the Jindu No. 1 and Cu Sha, the results showed that this gene only exists in the self-incompatible Cu Sha variety. In order to check if there are any differences in genomic sequence level between self-compatible varieties bulk and self-incompatible varieties bulk for *the HuS-RNase2* gene, the *HuS-RNase2* gene was amplified from the DNA of 20 different pitaya varieties (including 12 self-compatible varieties and 8 self-incompatible varieties), respectively. In the results from the agarose gel electrophoresis of PCR products shown in Figure 9C, there are no bands in self-compatible bulk (1–12) and there is one band in self-incompatible bulk (13–20), indicating that the gene was only present in self-incompatible varieties. Therefore, by detecting the genomes of each pitaya species, it can be preliminarily determined that *HuS-RNase2* is a candidate gynogenetic S gene associated with the SI trait of pitaya.

### 2.9. Screening for the DEGs Involved in Pollen–Stigma Interactions in Pollen

**Figure 10:**
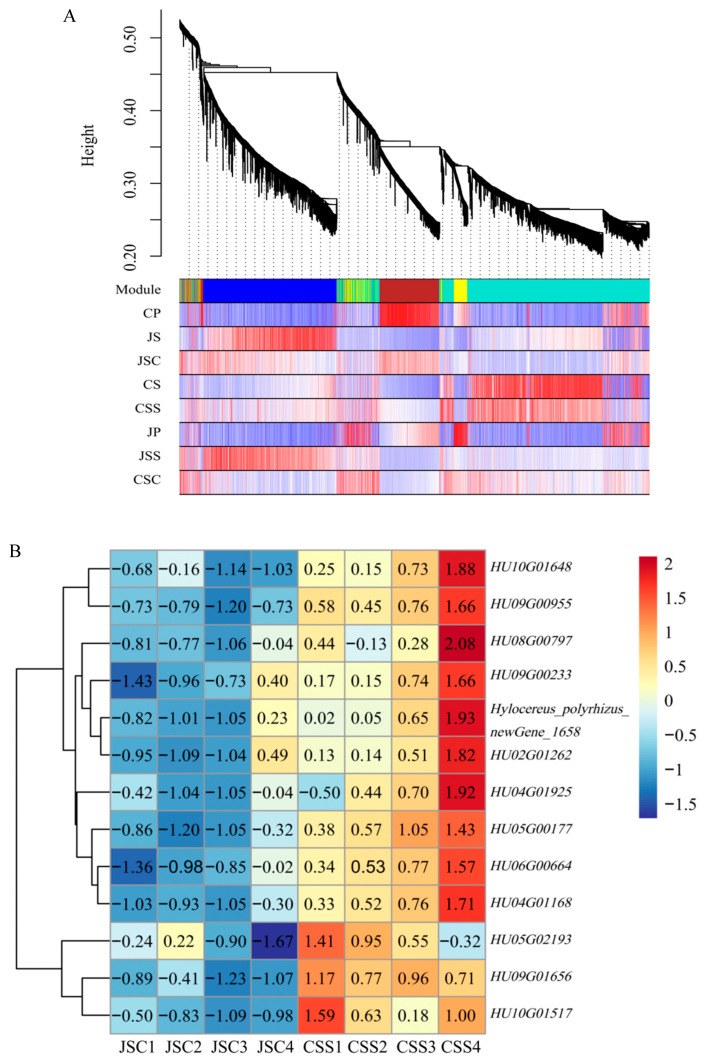
Upregulated expression of the DEGs in CP in response to the self-pollination group in Cu Sha. (**A**) The phylogram and correlation heat maps of the genes within each differential grouping. The first part is the clustered tree that was drawn by gene clustering, with branches representing genes; the second part is the gene clustering tree shown in different colors according to the different modules corresponding to the different clusters; and the third part is the heat map of the correlation between DEGs and modules—the darker the color, the higher the correlation between the genes in that module and that group. (**B**) The horizontal coordinate represents the sample name and the clustering result of the sample.

We constructed WGCNA for the DEGs in differential groupings (JP_vs_CP, JS_vs_CS, JSS_vs_CSS, and JSC_vs_CSC), thus determining whether the genes that were upregulated in the Cu Sha pollen were regulated due to their expression in post-pollination stigmas. A total of 4969 filtered genes (FPKM > 1) were clustered into four modules, in which CP was highly correlated with the brown module (*r* = 0.83, *p* = 0.01) (Figure 10A). We selected 189 upregulated DEGs in CP, of which 13 were upregulated in the Cu Sha (CSS1-4) after self-pollination, indicating that these genes may be involved in SI reactions (Figure 10B). These genes are mainly enriched in the molecular functional categories of GO terms, including binding, catalytic activity, structural activity, and antioxidant activity.

### 2.10. Screening and Analysis of the F-Box Genes Associated with SI

**Figure 11:**
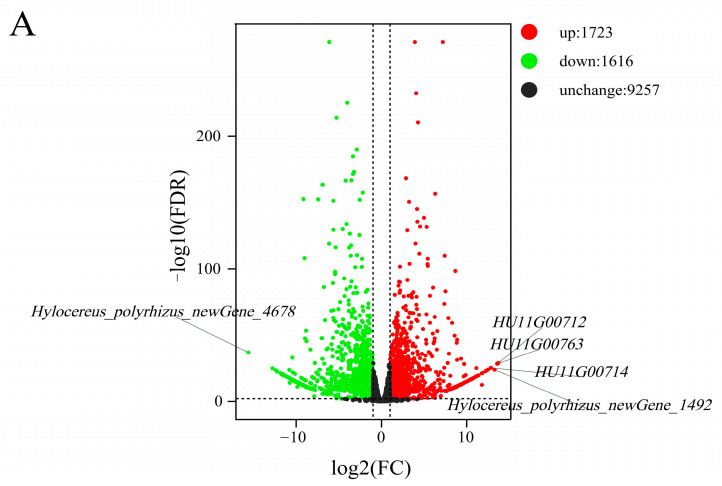

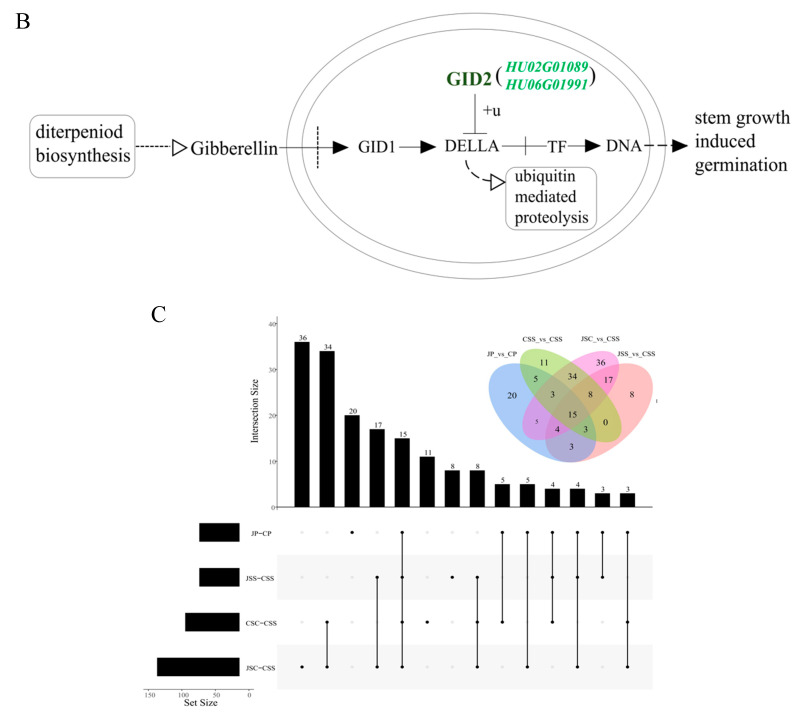
Dynamic changes in the *F-box* genes of the pollen and pollen–stigma interactions. (**A**) Volcano map of the differentially expressed *F-box* genes in the two types of pollen; each dot represents a gene and green and red dots represent down- and upregulated DEGs, respectively. The horizontal coordinates indicate the logarithmic value of the fold change in the expression of a gene in the two samples, and vertical coordinates indicate the negative logarithmic value of the statistical significance of the change in gene expression. The dots in the figure indicate the IDs of the five high-quality DEGs. (**B**) KEGG pathway maps of the downregulated expression of DEGs *HU02G01089* and *HU06G0199*. (**C**) The JSS_vs_CSS, JSC_vs_CSS, CSC_vs_CSS, and JP_vs_CP differential grouping Upset plots. The left side shows the original number of DEGs in each difference grouping, the top side shows the number of intersections by number from most to least, and the bottom side shows the intersection specifics.

In the differential grouping of the 2 types of pollen (JP_vs_CP), we screened 3339 DEGs (fold change ≥ 2, FDR < 0.01), 1723 upregulated expressed genes, and 1616 downregulated expressed genes (Figure 11A), of which the 58 DEGs were annotated as *F-box* were normalized for expression and then clustered for analysis (33 upregulated genes and 25 downregulated genes). The molecular functions in the GO item showed that these 58 genes are mainly involved in protein binding (GO:0005515) and ubiquitin–protein transferase activity (GO:0004842). These genes are also involved in protein processing of the endoplasmic reticulum, ubiquitin-mediated proteolysis, and plant hormone signal transduction, among other KEGG pathways. For example, the downregulated *F-box* genes *HU02G01089* and *HU06G01991* were annotated in the nr database as the F-box protein component of the GID2 protein, which is involved in the plant hormone signaling of the KEGG pathway. In addition, the GID2 protein acts as a component of the SCF, thus directly interacting with its DELLA protein (Figure 11B). The upregulated expression genes *HU11G01138* and *HU11G01139* and downregulated expression genes *HU02G00209*, *HU03G00273*, *HU03G01294*, and *HU10G01843* involved in ubiquitin-mediated proteolysis act as a target in the SCF complex recognizing subunit, and they participate in ubiquitination regulation after pollination.

To further investigate the functionality of the *F-box* in SI, we identified a total of 15 DEGs that were annotated to the F-box in each subgroup from JSS_vs_CSS, JSC_vs_CSS, CSC_vs_CSS, and JP_vs_CP (Figure 11C). There were 12 upregulated DEGs and 3 downregulated DEGs among these genes. Among them, 10 DEGs were enriched to ‘binding’ and ‘molecular function’ entries in the GO database, and 8 DEGs were enriched to ‘protein processing in endoplasmic reticulum’ and ‘plant-pathogen interaction’ pathways in the KEGG database.

### 2.11. qRT-PCR Validation of SI-Related DEG Genes

**Figure 12:**
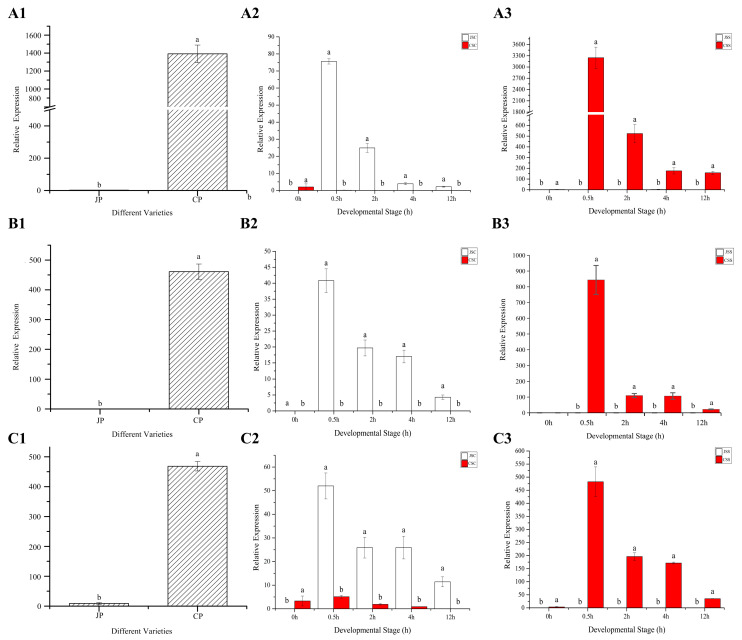

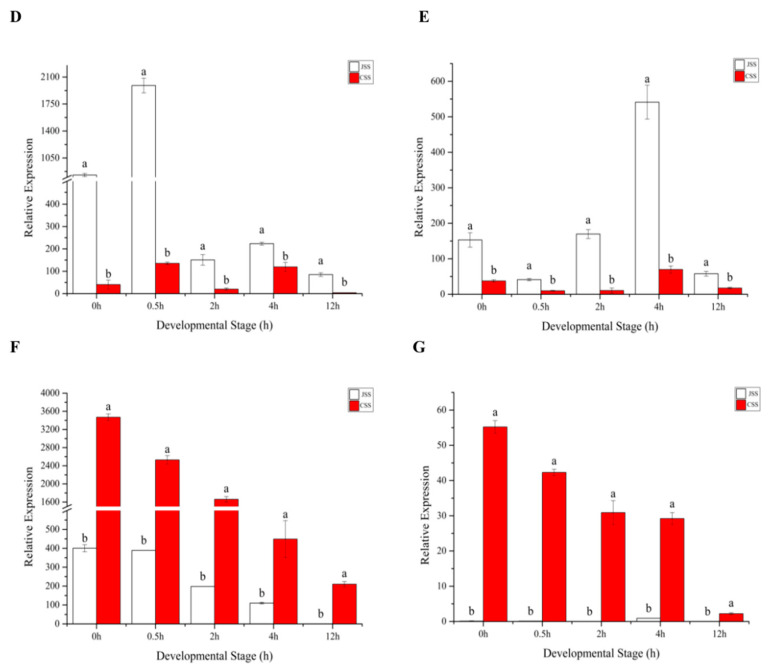
QRT-PCR results of the seven DEGs. (**A**–**C**) indicate the qRT-PCR results of *HU11G00714*, *HU11G00715*, and *HU11G00726*, respectively. (**D**–**G**) indicates the qRT-PCR results of *HU01G01021, HU04G01342, HU10G00362*, and *HU10G00364*, respectively. Different lowercase letters (a and b) indicate significant differences (*p* > 0.01).

The qRT-PCR results of three pollen *F-box* genes (*HU11G00714*, *HU11G00715*, and *HU11G00726*) associated with the SI trait in pitaya showed that the genes were abundantly expressed mainly in CP (Figure 12A1,B1,C1). After cross-pollination, the *F-box* genes in CP were highly expressed in Jindu No. 1, differing significantly from CSC (A2, B2, C2). Similarly, after self-pollination, this class of gene was positively responded to in the Cu Sha stigma (A3, B3, C3). The production results showed that self-pollination of the Cu Sha variety (CSS) was not fruitful, while self-pollination of the Jindu No.1 variety (JSS) was fruitful, indicating that, there may be proteins in the stigma of Jindu No.1 that collaborate with this class of *F-box* genes to finally complete fertilization. It is clear that this class of genes is abundantly expressed at 0.5 h during 0–12 h post-pollination, and that it gradually decreases with transcription time. The gene *HU01G01021*, which is annotated in the biological process: recognition of pollen (GO: 0048544) entry in the GO database, was significantly downregulated in the differential grouping of stigma samples (JSS_vs_CSS) at different time points after self-pollination from Jindu No. 1 and Cu Sha (Figure 12D), especially at 0.5 h after self-pollination. Thus, changes in gene expression may occur in a particularly short period of time during pollen–stigma interactions.

In this study, a large number of DEGs were annotated to the ‘ribosome’ entry of the KEGG pathway, from which we selected three proteins (HU04G01342, HU10G00362, and HU10G00364) to perform the analysis. The ribosomal inactivation protein HU04G01342 was significantly expressed in JSS (Figure 12E) and is involved in the defense response (GO:0006952) and negative regulation of translation (GO:0017148) in biological processes. HU10G00362 and HU10G00364 were enriched in the entries for the molecular functions of RNA binding (GO:0003723) and ribonuclease T2 activity (GO:0033897). This gene was significantly upregulated in self-pollinated Cu Sha stigmas (Figure 12F,G), and its expression decreased gradually with increasing pollination time.

## 3. Discussion

To identify the genes associated with SI in pitaya, as well as the key signal transduction factors involved in this response, we performed transcriptome sequencing and bioinformatics analysis on 60 samples from two tissues of pitaya, which were combined with cloning and the validation of key genes and qRT-PCR to comprehensively evaluate the two types of pollen and stigmas. We systematically analyzed the expression patterns and protein interactions of the DEGs before and after transcription in both self-pollination and cross-pollination scenarios. In addition, we also selected and analyzed the *S-RNase* and *F-box* genes that may be related to the SI trait of pitaya.

### 3.1. HuS-RNase2 May Be a Candidate Stamen S Gene Associated with the SI Trait of Pitaya

*S-RNase* is abundantly expressed in the style; in turn, it is secreted into the extracellular matrix of the style transport tissue. In the present study, we identified *HuS-RNase2* as a key protein in the two types of unpollinated stigmas that positively regulate SI response in pitaya. It was validated in the population, but also validated again after genome sequencing in all the selected varieties. Due to the inadequate annotation of the original genome, although the gene was a new gene after the splicing annotation, the amino acid sequence of the gene was found to be able to match exactly with the reference genome on chr11 at 77614890–77616360 via a comparison with the reference genome. However, it was finally annotated by nr annotation as S-locus-associated ribonuclease.

There is one conserved histidine residue for nuclease catalytic activity in each of the conserved C2 and C3 regions of S-RNase. Furthermore, if either histidine is mutated, it affects the ability of S-RNase [30]; therefore, nuclease activity plays a key role in the SI reaction. *S-RNase* is specifically expressed in the pistil, it mainly accumulates in the upper part of the style, enters the pollen tube, and degrades rRNA in large amounts, and it ultimately inhibits fertilization. However, rRNA was found to be degraded in self-incompatible pollen tubes by McClure et al. [31]. In the present study, most of the DEGs we identified were involved in ribosomal processes, such as the structural constituent of ribosome, including the *HU04G01342* identified in JSS_vs_CSS, *HU10G00362,* and *HU10G00364*, all of which were directly involved in the pollen–stigma recognition process. Thus, the inhibition of pollen tube elongation may not necessarily be solely controlled by the activity of S-RNase.

### 3.2. Possible Association of F-box with S-RNase in SI Reactions of Pitaya

In the GSI-type SI reaction, the S-locus F-box was thought to specifically recognize the hetero-me type S-RNase. In addition, it was also thought that multiple genes worked together to capture it and to label ubiquitin for its degradation, thus acting as a detoxification agent [21]. Qiao et al. (2004) used immunochemistry and Western blot techniques to localize the SLF protein in goldfish; then, the pollen antibody binding sites were found in the cytoplasm and periphery of the endoplasmic reticulum, while its product was still detected 16 h after pollen tube germination [32,33]. We identified 58 differentially expressed *F-box* genes in all the differential groups, which were annotated into multiple KEGG pathways such as binding and ubiquitination-mediated in the KEGG database. The three *F-box* genes validated by qRT-PCR were associated with the SI trait of pitaya, especially at 0.5 h after pollination, which is when the gene expression was most active. Although the expression level was significantly reduced at 12 h of pollination, it did not quench. Therefore, we can extend the pollination time and continuously observe the expression changes. Lai et al. (2002) used intra-S-site chromosome stepping to identify the pollen-specific expression of the encoded F-box protein (AhSLF-S2) from *Antirrhinum*, which mediates its GSI response only 9 kb away from the S2-RNase gene [15]. Therefore, we can localize the identified SLF protein to determine whether it interacts with *HuS-RNase2* and whether there is spatial and temporal variation, thus further analyzing the molecular mechanism of SI in pitaya.

### 3.3. Physiological Changes in Signal Transduction and Phytohormones in Pitaya

Of the 19,504 genes annotated to the KEGG database, most were annotated on the plant–pathogen interaction pathway. The SI response of plants may have some similarity with the signal transduction mechanism of pathogen–plant interactions, and their defense mechanisms may have a common ancestor. As invaders, both pollen and pathogenic bacteria are extracellular material, which the host recognizes and thus determines whether to allow them to invade the cell and develop into spore-like structures [34]. Similarly, there are a large number of differential transcripts involved in environmental information processing. Within 0.5 h of pollination, there were significant changes in the number of DEGs in both self-pollination and hetero-pollination scenarios, indicating that—after pollen–stigma contact—the signal transduction factors or hormones change dramatically at the transcriptional level. For example, HU03G01065 belongs to an auxin-induced protein, which is involved in tryptophan metabolism on the KEGG pathway plant hormone signal transduction (ko04075). Ubiquitination mediates AUX/IAA synthesis, thus controlling the cell expansion that impedes reproductive development and is upregulated in CSS. *HU07G00012* interacts as a calmodulin-binding receptor-like cytoplasmic kinase, and *HU07G00813* is a serine/threonine-protein kinase CTR1 that downregulates the expression in SIs. Further study of this class of enriched genes may reveal genes that are essential for the pollen–stigma signaling pathway.

### 3.4. SI Reaction Develops in a Short Period of Time

The recognition of pollen to the stigma is a particularly rapid process. Observations on the development of pollen tubes after cross-pollination and self-pollination in pitaya show that when pollen falls on the stigma, pollen wall proteins can be released within a few minutes. The pitaya flower withered at about 12 h when the pollen tube had elongated by at least 1 cm in the style. In this study, most of the highly expressed differential genes reached their peak expression on the stigma after 0.5 h of pollination, and the expression gradually decreased as the transcription time increased (to such an extent that the expression was almost zero at 12 h). Therefore, the occurrence of the SI response in pitaya was established in a short time, which is also consistent with the results of previous studies.

## 4. Materials and Methods

**Figure 13:**
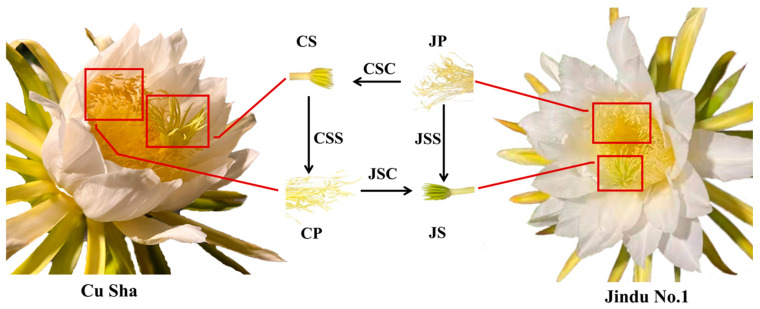
Sample collection plots of Cu Sha (self-incompatible) and Jindu No. 1 (self-compatible) with CS and JS as unpollinated, CP and JP as pollen, CSS and JSS as self-pollinated, and CSC and JSC as cross-pollinated stigmas.

In this study, the experimental material of pitaya was prepared by the bagging method, and the material was obtained from Dongfang City (108.65° E, 19.10° N), Hainan Province, China. Artificial pollination was carried out at night in favorable weather conditions, with self-pollination and hetero-pollination performed for the self-compatible Jindu No. 1 (*H. polyrhizus*) and self-incompatible Cu Sha (*H. undatus*) stigmas, respectively. For both types, pollen samples were collected before pollination and at different time intervals after pollination. The entire stigma and a 1 cm pollen tube section under the stigma were collected for each sample (collections were made before pollination and at 0.5, 2, 4, and 12 h after self-pollination or cross-pollination (orthogamy and antigamy)) (Figure 13). “J/C” denotes the pitaya varieties of “Jindu No.1” and “Cu Sha”; “S/P” denotes the collection of “stigma” and “pollen”; S/C denotes “self-pollination” and “cross-pollination”; 1/2 /3/4 denotes the four periods after pollination “0.5, 2, 4 and 12h”; and, finally, the samples were named by “species-tissue-pollination method-pollination period”. Three replicate samples of all the treatments were rapidly frozen in liquid nitrogen and stored in a −80°C refrigerator (Supplementary Materials: Table S6).

### 4.1. Total RNA Extraction cDNA Library Construction

The total RNA was extracted from the pitaya stigmas and pollen using a modified CTAB method. The pitaya tissues had enough polysaccharides and polyphenols; therefore, in this study, we modified the CTAB method for extracting plant RNA efficiently by using phenols in the extraction process. Three replicates of each sample were used. RNA purity and concentration were determined with an ultra-micro spectrophotometer (NanoPhotometer^®®^ N50, Munich, Germany), and the quality of the RNA was checked via 3% agarose gel electrophoresis [35]. The cDNA library was constructed. The procedures were as follows: mRNA was isolated by Oligo(dT)-attached magnetic beads and then randomly fragmented in a fragmentation buffer. First-strand cDNA was synthesized with fragmented mRNA as a template and random hexamers as primers, followed by second-strand synthesis with the addition of PCR buffer, dNTPs, RNase H, and DNA polymerase I. Purification of cDNA was processed with AMPure XP beads. Double-strand cDNA was subjected to end repair. Adenosine was added to the end and ligated to the adapters. AMPure XP beads were applied here to select the fragments within a size range of 300–400 bp. Finally, the cDNA library was obtained via certain rounds of PCR on the cDNA fragments.

### 4.2. Transcriptome Sequencing

The pooled cDNA libraries were sequenced using the Illumina high-throughput sequencing platform, which is based on synthetic sequencing (SBS) technology, and filtered through a series of quality controls to obtain clean data [36]. HISAT2 (high-efficiency comparison system) was used for sequence comparison with the specified reference genome (http://www.pitayagenomic.com/download.php (accessed on 5 June 2022)) [37]. Mapped reads were spliced using StringTie [38], and the selective splicing events present in individual samples were obtained by ASProfile software [39]. The 9167 novel genes uncovered were sequenced against nr [40], SwissProt [41], GO [42], COG [43], Pfam [44], and KEGG [45] databases using BLAST software, and the KEGG Orthology results of the novel genes were obtained using KOBAS 2.0. After predicting the amino acid sequences of the new genes, HMMER software [46] was used to compare them with the Pfam database in order to obtain annotation information for the 2935 new genes.

### 4.3. Identification of Gene Expression Level and DEGs

The fragments per kilobase of transcript per million fragments mapped (FPKM) method was used to measure the gene expression levels. The 60 samples were subjected to principal component analysis (PCA). The stigmas and pollen before pollination and after (0.5, 2, 4, and 12 h) self-pollination, heterogeneous pollination, the different varieties that were used as differential groups, and DESeq2 were all used for the differential expression analysis between the sample groups [47,48]. A fold change of ≥2 and a false discovery rate (FDR) of <0.01 were used as the screening criteria and are shown in volcano plots.

### 4.4. Clustering Analysis and Construction of the Gene Co-Expression Network

We constructed a weighted correlation network analysis (WGCNA) in which each node within all the samples of JS, CS, JSC, CSC, JSS, and CSS was defined as one gene. To ensure an effective linkage between the key genes, we set—via weighting—a module similarity threshold of 0.25 for the screen expression correlations, selected k-means of >0.7 as the module members, and the minimum number of genes within the module was set to 30. In screening the upregulated genes in CP for the regulation of corresponding genes in the column headers, we adopted the same WGCNA for the DEGs within each differential grouping.

### 4.5. Enrichment Analysis of DEGs Sets in Different Pollination Modes

To determine the common expression patterns of DEGs in the different pollination types, we conducted a gene set enrichment analysis (GSEA). The distribution of all the genes that were not screened by log2FC and FDR in each differential grouping is reflected in the heat maps. We used the detection of the differential usage of exons from RNA-seq data (DEXSeq) for differential exon usage (DEU) analysis, using an FDR of <0.01 as a screening for differential exon expression. The location of the region and the density on the 11 chromosomes of the pitaya genome were localized, and circos circle maps were produced for the purpose of visualization.

### 4.6. Co-Expression Trend of Genes at Different Transcription Stages

To find the co-expression trends of genes within stigmas at different transcriptional periods in pitaya, we took the differential genes between JS_vs_CS (fold change ≥ 2, FDR < 0.01) as the initial value and log2 (fpkm + 1) for the differential gene expression at different periods after pollination for both stigmas. This was achieved by using Euclidean distance as the distance metric, and k-means clustering was performed to determine the different patterns of the mRNA expression changes between the samples.

### 4.7. Interacting with SI-Response-Related Gene Protein Networks

To elucidate the molecular mechanisms and key regulatory proteins involved in SI and fertilization, we screened the shared DEGs of JSS_vs_CSS and CSC_vs_CSS using a fold change of ≥2 and an FDR of <0.01 as the criteria. The screened protein interaction network data files were directly imported into Cytoscape software for visual editing [49].

### 4.8. Functional Identification and Population Validation of HuS-RNase2

We blasted the amino acid sequence of the screened *HuS-RNase2* on NCBI to determine whether it contains a region that is highly homologous to ribonuclease T2. The forward and reverse primer sequences of transcript *HuS-RNase2* are as follows: F: atggccatggaggccgaattcGGAAAAATGAAAAAAGTTTGGTTGC; R: ccgctgcaggtcg acggatccGCGCAATTGA AATTTGTGGTAA. The DNA of the 20 pitaya stems was extracted and used for the population validation of *HuS-RNase2*.

### 4.9. qRT-PCR Validation

Eight DEGs were selected to verify, via the qRT-PCR technique, the reliability of the RNA-Seq data. Gene-specific primers were designed by Primer Premier 5.0 software (Supplementary Materials: Table S7). UBQ genes were selected as internal reference genes. High-quality RNA was reverse transcribed using HiScript III RT SuperMix for qPCR (+ gDNA wiper) (Vazyme, Nanjing, China), and the mRNA was analyzed using ChamQ Universal SYBR qPCR Master Mix (Vazyme, Nanjing, China) on the ABA7500 Real-Time. The relative expression levels of mRNAs were determined in the ABA7500 real-time PCR system according to the manufacturer’s requirements. The expression of DEGs was calculated using the 2^ΔΔCT^ method [50]. The significance of expression differences was analyzed using SPSS software, and then histograms were created using GraphPad.

## 5. Conclusions

Here, we report the comprehensive analysis of the transcriptome sequence information related to the SI response of pitaya. We evaluated the gene expression patterns in the pollen and stigma of pitaya with two different types. DEGs associated with SI traits in pitaya were mainly annotated to the ribosomal, ubiquitin-mediated, and signal transduction entries of various databases. In addition, we identified *HuS-RNase2* as a candidate stamen S gene for the SI trait in pitaya. These findings will help the efforts to further understand the molecular mechanism of the SI trait in pitaya, and they will offer a reference for developing new SI varieties.

## Figures and Tables

**Table 1:** Statistics on the number of DEGs between the different groups.

Comparison Group	0.5 h	2 h	4 h	12 h
JSS_vs_CSS	4463	4099	3649	3988
JSS_vs_JSC	2913	6597	7580	6129
CSC_vs_CSS	4016	6288	3540	7970
JSC_vs_CSC	2821	3222	4214	2252

## Data Availability

All data generated and analyzed during the study are included in the published article and its Supplementary Information files. Other data is temporarily not disclosed.

## Supplementary Materials

The following supporting information can be downloaded at: https://www.mdpi.com/article/10.3390/ijms241411406/s1.
