# Supplementary material for: The Pitaya Flower Tissue’s Gene Differential Expression Analysis between Self-Incompatible and Self-Compatible Varieties for the Identification of Genes Involved in Self-Incompatibility Regulation
Source: Int J Mol Sci. 2023 Jul 13;24(14):11406. doi: 10.3390/ijms241411406 (PMC10379629; doi:10.3390/ijms241411406)
Supplement: Supplementary file 1 [file ijms-24-11406-s001.zip › Figure S1 Annotation of gene KEGG pathway in K8.pdf]

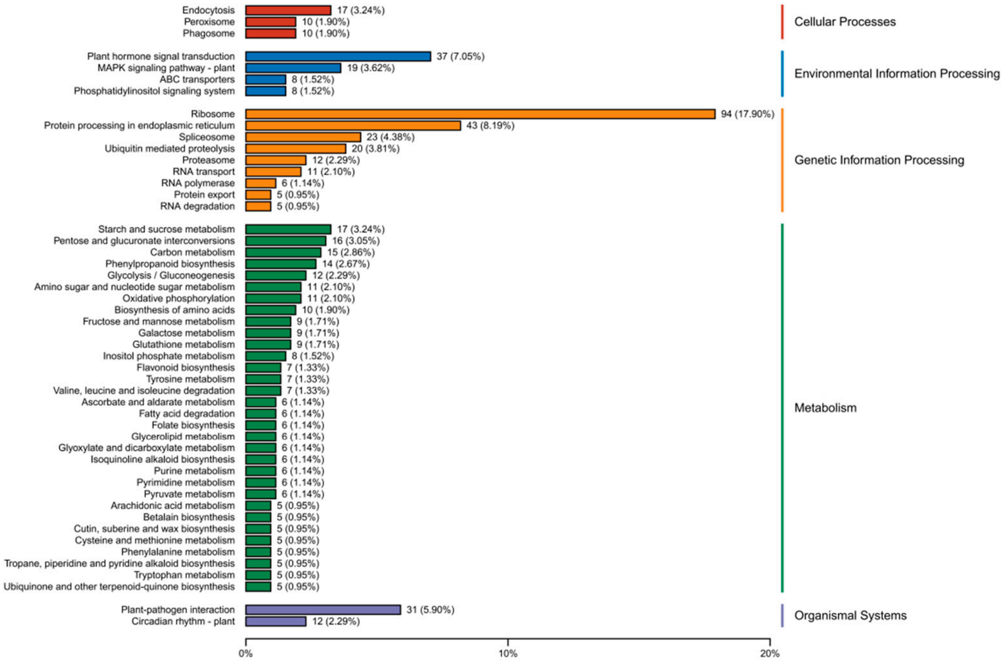

Figure S1 Annotation of gene KEGG pathway in K8. Y-axis: KEGG pathway terms; X-axis: Number and the percentage of genes annotated to the KEGG pathway.
