# Supplementary figures and images for: The Pitaya Flower Tissue’s Gene Differential Expression Analysis between Self-Incompatible and Self-Compatible Varieties for the Identification of Genes Involved in Self-Incompatibility Regulation
Source: Int J Mol Sci. 2023 Jul 13;24(14):11406. doi: 10.3390/ijms241411406 (PMC10379629; doi:10.3390/ijms241411406)

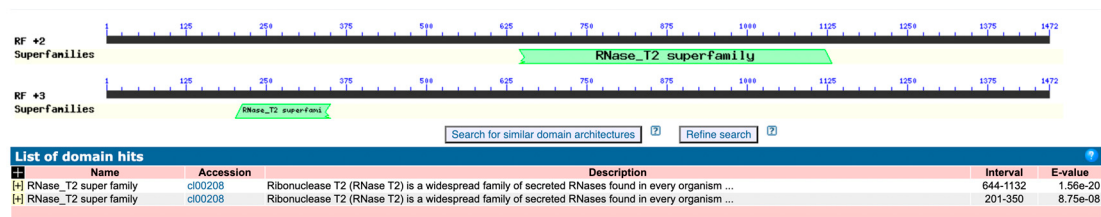

Figure S2 Conserved region of S-RNase2. The presence of two RNase-T2 regions in S-RNase2.

Supplement: Supplementary file 1 [file ijms-24-11406-s001.zip › Figure S2 Conserved region of S-RNase2.pdf]
